# The puzzling pathophysiology of frozen shoulders – a scoping review

**DOI:** 10.1186/s40634-020-00307-w

**Published:** 2020-11-18

**Authors:** T. Kraal, J. Lübbers, M. P. J. van den Bekerom, J. Alessie, Y. van Kooyk, D. Eygendaal, R. C. T. Koorevaar

**Affiliations:** 1 Department of Orthopaedic Surgery, Spaarne Gasthuis, Hoofddorp, the Netherlands; 2 Haarlem, The Netherlands; 3 Department of Molecular cell biology and Immunology, Amsterdam University Medical Centre, Amsterdam, the Netherlands; 4 Department of Orthopaedic Surgery, OLVG, Amsterdam, the Netherlands; 5 Avans University of Applied Science, Breda, The Netherlands; 6 Department of Orthopaedic Surgery, Amsterdam University Medical Centre, Amsterdam, the Netherlands; 7 Department of Orthopaedic Surgery, Deventer Hospital, Deventer, the Netherlands

**Keywords:** Shoulder, Frozen shoulder, Adhesive capsulitis, Stiffness, Pathophysiology, Histology, Etiology

## Abstract

**Purpose:**

The pathophysiology of frozen shoulders is a complex and multifactorial process. The purpose of this review is to scope the currently available knowledge of the pathophysiology of frozen shoulders.

**Methods:**

A systematic search was conducted in Medline, Embase and the Cochrane library. Original articles published between 1994 and October 2020 with a substantial focus on the pathophysiology of frozen shoulders were included.

**Results:**

Out of 827 records, 48 original articles were included for the qualitative synthesis of this review. Glenohumeral capsular biopsies were reported in 30 studies. Fifteen studies investigated were classified as association studies. Three studies investigated the pathophysiology in an animal studies. A state of low grade inflammation, as is associated with diabetes, cardiovascular disease and thyroid disorders, predisposes for the development of frozen shoulder. An early immune response with elevated levels of alarmins and binding to the receptor of advance glycation end products is present at the start of the cascade. Inflammatory cytokines, of which transforming growth factor-β1 has a prominent role, together with mechanical stress stimulates Fibroblast proliferation and differentiation into myofibroblasts. This leads to an imbalance of extracellular matrix turnover resulting in a stiff and thickened glenohumeral capsule with abundance of type III collagen.

**Conclusion:**

This scoping review outlines the complexity of the pathophysiology of frozen shoulder. A comprehensive overview with background information on pathophysiologic mechanisms is given. Leads are provided to progress with research for clinically important prognostic markers and in search for future interventions.

**Level of evidence:**

Level V.

## Introduction

Frozen Shoulder (FS) is a common cause of shoulder pain associated with restricted active and passive range of motion. Although this condition has been recognized as a clinical disease entity for about 150 years, we still have not unraveled the pathophysiology yet. FS has often been described as a self-limiting condition, with recovery within two to three years for the majority of patients [1]. However, symptoms of mild to moderate pain and stiffness are reported in 27–50% of patients at long term [2–4]. Even in patients with a favorable natural course of the condition, there is still an extensive period to deal with pain, and functional limitations.

Current surgical interventions, such as manipulation under anesthesia or arthroscopic capsular release, are aimed at the advanced stage of the disease, when the fibrotic cascade has already had its effect. To optimize treatment the treatment of FS, it is of fundamental importance to get a better understanding of the pathophysiology. With advancing knowledge, it might become possible to intervene early on in the disease process.

The aim of this scoping review is to systematically collate the currently available knowledge that we have about the pathophysiology of FS. The histologic findings and the mechanism of tissue fibrosis on a cellular level are addressed. The purpose is to give and apprehensible overview which aids clinicians in the understanding of the pathophysiology and to translate this to clinical implications.

## Materials and methods

A systematic search in Medline, Embase and the Cochrane library was conducted in all three databases on the fifth of October 2020. The search was build including the following terms; “frozen shoulder”, or (“shoulder” AND “adhesive capsulitis”), “pathophysiology”, (“etiology” or “aetiology”) and (“histology” or “anatomy and histology”). Publications had to be original papers published in English after the first of January 1994. The limit of 1994 was chosen since the techniques to analyze tissue samples of more than 25 years ago are most likely outdated and therefore not relevant anymore. Articles were eligible for inclusion if the there was a substantial focus on the pathophysiology of FS. All studies on tissue samples from FS patients were eligible for inclusion. Association studies between medical co-morbidities and FS were only eligible if the pathophysiologic mechanism between the investigated condition and FS was discussed. Basic science studies (in vitro or animal model studies) were eligible for inclusion if the aim of the article was to clarify the pathophysiology of FS. Reviews, case reports and imaging studies were excluded.

## Results

**Fig. 1:**
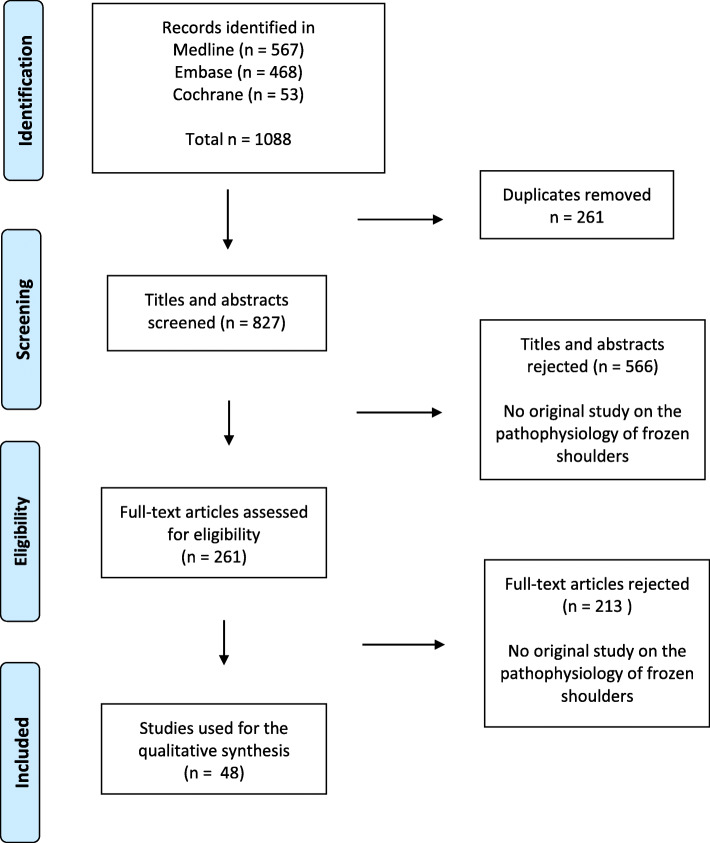
PRISMA flow diagram

A number of 1088 potential relevant studies were identified in the searches. After removal of duplicates, titles and abstracts were screened from a total of 827 studies. A low threshold was used to verify if the full text articles included unique or relevant information on the pathophysiology of FS. This resulted in 48 original studies eligible for inclusion in the qualitative synthesis of this review. A PRISMA flow chart of the review process is presented in Fig. 1. (Fig. 1).

The 48 included articles are categorized by study design in three tables, in a chronological order. The most relevant finding for each article is given. Table one shows all 30 original articles wherein tissue samples from the glenohumeral joint were analyzed. These are mostly case control studies with a small number of patients. The controls were usually patients undergoing arthroscopy for different shoulder pathology like instability or rotator cuff surgery. The number of FS patients, controls, biopsy location and used method for tissue analysis is described for each study.(Table 1) Table two shows 15 association studies wherein the pathologic mechanism between a certain co-morbidity (e.g. diabetes, thyroid disorder) and FS is discussed. This includes studies investigating the association between FS and serum levels in peripheral blood, for example hormones, lipids or gene polymorphism.(Table 2) Table three displays three animal (rats) studies investigating the pathophysiologic pathways in FS in detail. (Table 3).

**Table 1:** **Biopsy studies;** studies investigating the pathophysiology of frozen shoulder with glenohumeral capsular tissue samples

Author	Year	Study design	Biopsy location	***n*** FS	***n*** Controls	Analysis method	Most relevant findings
Hannafin [5]	1994	case series	anterior, inferior and posterior	15	–	histology	Frozen shoulder starts with a hypervascular synovitis followed by diffuse fibroplasia with thickening and contracture of the capsule
Bunker [6]	1995	case series	CHL + RI	12	–	immunohistochemistry	Active fibroblastic proliferation with differentiation into myofibroblasts and the deposition of thick nodular bands of collagen
Rodeo [7]	1997	case control	anterosuperior	19	21	immunohistochemistry	Hypervascular synovial hyperplasia with fibroblasts, occasional T-cells, B-cells and newly synthesized collagen type I and III was found. TGF-β, PDGF, IL-1β and TNF-α are involved in an inflammatory and fibrotic process in frozen shoulders
Bunker [8]	2000	case serie	RI	14	4	RT-PCR	The presence of mRNA for a large number of cytokines and growth was demonstrated in frozen shoulder capsular tissue
Ryu [9]	2006	case control	RI	11	5	immunohistochemistry, western blot	Immunostaining for VEGF was stronger in frozen shoulders compared to controls
Hand [10]	2007	case series	RI	22	–	immunohistochemistry	Fibroblastic proliferation and an infiltrate of chronic inflammatory cells (mast cells, T cells, B cells and macrophages) was found
Kilian [11]	2007	case control	RI	6	6	immunohistochemistry, RT-PCR	Significant enhancement of α-1(I) mRNA transcription (mature collagen) was found
Uhthoff [12]	2007	case series	5 different locations	4	–	immunohistochemistry	Fibroplasia together with type III collagen was present in the entire joint capsule. Contracture, (vimentin expression), was found only in the anterior joint capsule (rotator interval and CHL)
DePalma [13]	2008	case series	capsule	32	–	histology	Evidence of a low grade chronic inflammatory process with variable involvement of the biceps tendon sheath was found
Kanbe [14]	2009	case series	RI	10	–	immunohistochemistry	NF-κB, IL-6, MMP3, β1-integrin and VEGF were expressed in the synovial tissue of frozen shoulders
Li [15]	2009	case control	RI	12	12	RT-PCR	A higher expression of mRNA for TGF-β and several MMPs was found
Kabbabe [16]	2010	case control	4 different locations	13	10	qPCR	Inflammatory (IL-6 and IL-8) and fibrogenic (MMP3) cytokines were expressed at a higher level in frozen shoulders compared to controls
Nago [17]	2010	case series + in vitro cell culture	RI	7	–	histology, RT-PCR	Treatment of cultured glenohumeral/synovial fibroblast from frozen shoulder patients with hyaluronan inhibited cell proliferation and expression of adhesion related procollagens and cytokines.
Hagiwara [18]	2012	case control	RI + MGHL + IGHL	12	18	immunohistochemistry, qPCR, scanning acoustic microscopy	A higher number of cells, stiffer capsular tissue and increased gene expression related to fibrosis (COL1A1, PDGF-B) inflammation (IL-1β) and chondrogenesis was found
Xu [19]	2012	case control	RI	8	10	immunohistochemistry	Increased expression of nerve growth factor receptor and new nerve fibers were found in frozen shoulder capsular tissue compared to controls
Kim [20]	2013	case series	RI	17	9	immunohistochemistry, RT-PCR	ICAM-1 was increased in capsular tissue, synovial fluid, and serum of frozen shoulder patients compared to controls
Lho [21]	2013	case control	RI + subacromial bursa	14	7	immunohistochemistry, RT-PCR, ELISA	IL-1α, IL-1β, TNF-α, COX-1 and COX-2 were expressed at higher levels in joint capsule of frozen shoulder patients compared to controls. In the subacromial bursa, IL-1α, TNF-α and COX-2 were expressed at higher levels
Raykha [22]	2014	case control + in vitro cell culture	RI	?	?	western blot, RT-PCR	β-catenin and IGF-2 expression were found to be elevated in frozen shoulders compared to controls
Cho [23]	2015	case control	capsule	18	18	immunohistochemistry, RT-PCR	Upregulation of acid sensing ion channels (ACICs)was found in capsular tissue and synovial fluid of frozen shoulder patients
Cohen [24]	2016	case control	anteroinferior capsule	9	8	RT-PCR	Elevated expression of Tenascin C and Fibronectin 1 mRNA was found in capsular tissue of frozen shoulder patients.
Hettrich [25]	2016	case control	anterior and posterior	20	14	immunohistochemistry	Intra articular corticosteroid injection reduces fibrosis, vascular hyperplasia and myofibroblast differentiation
Hwang [26]	2016	case control	RI	8	14	immunohistochemistry	Immunoreactivity of AGEs was stronger in frozen shoulder capsules compared to controls
Cui [27]	2017	case control	capsule + bursa + synovium	5	2	RNA sequencing	147 genes were upregulated and 24 downregulated in capsular tissue of frozen shoulder patients compared to controls
Cher [28]	2018	case control	RI	10	10	immunohistochemistry	Immunoreactivity of alarmins was stronger in frozen shoulder patients. The expression of the alarmin HMGB1 correlated with the severity of pain
Hagiwara [29]	2018	case control	RI + MGHL + IGHL	12	7	shotgun proteome analysis	The pathophysiology might differ between the upper and lower parts of the joint capsule. In the RI and MGHL samples, different proteins were higher expressed compared to the IGHL samples
Akbar [30]	2019	case control + in vitro cell culture	RI	10	10	immunohistochemistry, qPCR, ELISA	Fibroblasts in FS have activated phenotype with an increased expression of fibroblast activation markers. Cultured FS fibroblasts produced elevated levels of inflammatory proteins (IL-6, IL-8, CCL-20)
Cho [31]	2019	case control + animal (rat) study	capsule	21	13	immunohistochemistry	Overexpression of IL-6, MMP-2 and MMP-9 may be associated with frozen shoulder
Kamal [32]	2020	case control	anterior	22	26	RT-PCR	Inflammation and ECM remodelling were the most signifant and highly enriched processes in frozen shoulder. MMP13 expression was increased and TNF-α expression was reduced in frozen shoulders
Yang [33]	2020	case control + in vitro cell culture	RI	9	10	immunohistochemistry, RT-PCR, flow cytometry	COL1A1, COL3A1, TGF-β1, and IL-6 were expressed at increased levels in the frozen shoulder group compared to controls. The presence of calcitonin receptors in shoulder capsular tissue was confirmed. Treatment with salmon calcitonin decreased the expression of COL1A1, COL3A1, fibronectin 1, laminin 1, TGF-β1 and IL-1α
Yano [34]	2020	case control	CHL + IGHL	33	25	immunohistochemistry, RT-PCR, high performance liquid chromatography	AGEs and HMGB1 might play important roles in the pathogenesis of frozen shoulder. Gene expression levels of RAGE, HMGB1, TLR2, TLR4 and NF-κB were significantly greater in frozen shoulders compared to controls

*CHL* coracohumeral ligament, *RI* rotator interval, *MGHL* middle glenohumeral ligament, *IGHL* inferior glenohumeral ligament, *RT-PCR* real time polymerase chain reaction, *ELISA* enzyme linked immune sorbent assay, *TGF-β* transforming growth factor beta, *AGE* advanced glycation end product, *MMP* matrix metalloproteinase, *TIMP* tissue Inhibitor of Metallo Proteinases, *TSH* thyroid stimulating hormone, *IGF* insulin like growth factor, *ICAM* intercellular adhesion molecule-1, *ECM* extracellular matrix, *TNF-α* tumor necrosis factor alfa, *VEGF* vascular endothelial growth factor

**Table 2:** **Association studies;** studies investigating the association between frozen shoulder and a co-morbidity, focussed on the pathophysiologic mechanism

Author	Year	Study Design	***n*** FS	***n*** controls	analysis method	Most relevant findings
Bunker [35]	1995	case series	43	43	peripheral blood samples	Fasting serum triglyceride and cholesterol levels were significantly elevated in frozen shoulder patients
Mullet [36]	2007	case control	15	15	glenohumeral joint aspirate, in vitro cell culture	Proliferation of cultured human fibroblast cells was significantly increased by stimulation of growth factors from joint aspirate of frozen shoulder patients
Lubis [37]	2013	case control	50	50	peripheral blood samples	MMP1 and MMP2 levels were significantly lower, while TIMP1, TIMP2 and TGF-β1 were higher in frozen shoulder patients compared to controls
Austin [38]	2014	case control	150	NHANES nationwide study	patient chart review	A relationship is suggested between systemic inflammation with hyperglycaemia and hypertension and frozen shoulder
Huang [39]	2014	cohort	162	Longitudinal health insurance database	ICD-9-CM codes	Hyperthyroid patients have a 1.22 fold higher risk to develop frozen shoulder compared to the general population in Taiwan
Sung [40]	2014	case control	300	900	peripheral blood samples	Hypercholesterolemia, and inflammatory lipoproteins have a significant association with frozen shoulder
Booker [41]	2017	case control	20	26	capsular biopsies for microbiological culture	No correlation was found between the incidence of P. Acnes and frozen shoulder
Chan [42]	2017	retrospective cohort	197	24,220	peripheral blood samples	Cumulative HbA1c was (dose dependent) associated with an increased incidence adhesive capsulitis
Chen [43]	2017	case control	42	50	peripheral blood samples - ELISA	IL-1β was expressed at higher levels in frozen shoulder patients and is associated with susceptibility of frozen shoulder
Holte [44]	2017	case control	100	73	skin biopsies - liquid chromatography mass spectometry	Joint stiffness was associated with long term HbA1c and AGEs
Schiefer [45]	2017	case control	93	151	peripheral blood samples	Hypothyroidism was significantly more prevalent in frozen shoulder patients than in controls. A correlation between TSH levels with the severity of frozen shoulders was suggested
Gumina [46]	2018	prospective observational	27	genome database	peripheral blood samples - PCR	APO-A1-G75A lipoprotein polymorfism was found as a risk factor for the severity of frozen shoulder
Kalson [47]	2018	cohort	549	5989 (Twins UK registry)	qPCR	Frozen shoulder patients had a significant relation with telomere length. It is suggested that telomere repair defects contribute to joint fibrosis
Park [48]	2018	case control	37	222	peripheral blood samples	Inflammatory lipoproteins are associated with adhesive capsulitis accompanied by diabetes
Cohen [49]	2019	case control	186	600	peripheral blood samples - genotyping	Certain genetic variants, SNPs of MMP13, MMP 9 and TGFβ1 were identified as independent risk factors for frozen shoulder

*PCR* polymerase chain reaction, *ELISA* enzyme linked immune sorbent assay, *TGF-β* transforming growth factor beta, *AGE* advanced glycation end product, *MMP* matrix metalloproteinase, *TIMP* tissue Inhibitor of Metallo Proteinases, *TSH* thyroid stimulating hormone, *SNP* single nucleotide polymorphism, *IL-1β* Interleukin-1β

**Table 3:** **Animal studies;** animal studies with the specific aim to investigate the pathophysiology of frozen shoulder

Author	Year	Study Design	Method used for analysis	Most relevant findings
Watson [50]	2011	animal model (rats)	RT-PCR	TGF-β1 gene transfer induced a fibrotic condition comparable to frozen shoulder patients with similar expression levels of ECM proteins, MMPs, adhesion- and collagen proteins
Xue [51]	2016	animal model (rats) + cell culture	RT-PCR and gene silencing with a lentivirus	Smad4 silencing can suppress chronic inflammation and fibrosis in joint tissue by inhibiting the TGF-β/Smad pathway
Blessing [52]	2019	animal model (rats) + cell culture	immunohistochemistry	Local delivery of Relaxin-2 downregulates type I collagen and α-smooth muscle actin production

*CHL* coracohumeral ligament, *RI* rotator interval *RT-PCR* real time polymerase chain reaction, *TGF-β* transforming growth factor beta, *ECM* extracellular matrix, *MMP* matrix metalloproteinase, *α-SMA* α - smooth muscle actin

## Patho-anatomy

The restriction in passive range of motion in FS is caused by a contracted glenohumeral capsule. The normal shoulder joint has a volume of at least 15 ml, and on average 20 ml [53]. In FS, the joint volume can be less then 5 ml [54]. Capsular stiffness is demonstrated in studies measuring intra-articular pressure while distending the capsule. Pressure volume curves show a much steeper rise in FS compared to controls and capsular rupture occurs in FS at a much lower volume with higher pressures compared to normal shoulders [55–57]. It has long been hypothesized that the rotator interval with the coracohumeral ligament (CHL) is involved in the pathophysiologic process of FS, and might have a pivotal role in the development of FS, and the rest of the joint capsule is involved later on in the process [58–61]. The CHL spans the extra-articular side of the rotator interval, is strained in external rotation, and release of the CHL is an important part of the surgical release of a FS [62, 63]. Several other findings are reported in the literature that support a prominent role in the etiology of FS for the rotator interval. Ultrasound guided corticosteroid injections in the rotator interval and around the CHL had greater effect on pain and range of motion compared to intra-articular corticosteroid injections directed from posterior [61]. Fluorodeoxyglucose (FDG)-PET CT scans in FS demonstrate that FDG uptake is predominantly located in the rotator interval, anterior joint capsule and axillary recess [64]. Angiography studies identified neovascularization, branching of the thoracoacromial artery, in the rotator interval of FS patients [65]. Upregulation of proteins involved in collagen metabolism, cell adhesion and the immune response were identified in the rotator interval of FS patients [29]. The gliding mechanism of the biceps tendon sheat, the lateral border of the rotator interval, was involved to a variable degree [66].

## Histologic findings

**Fig. 2:**
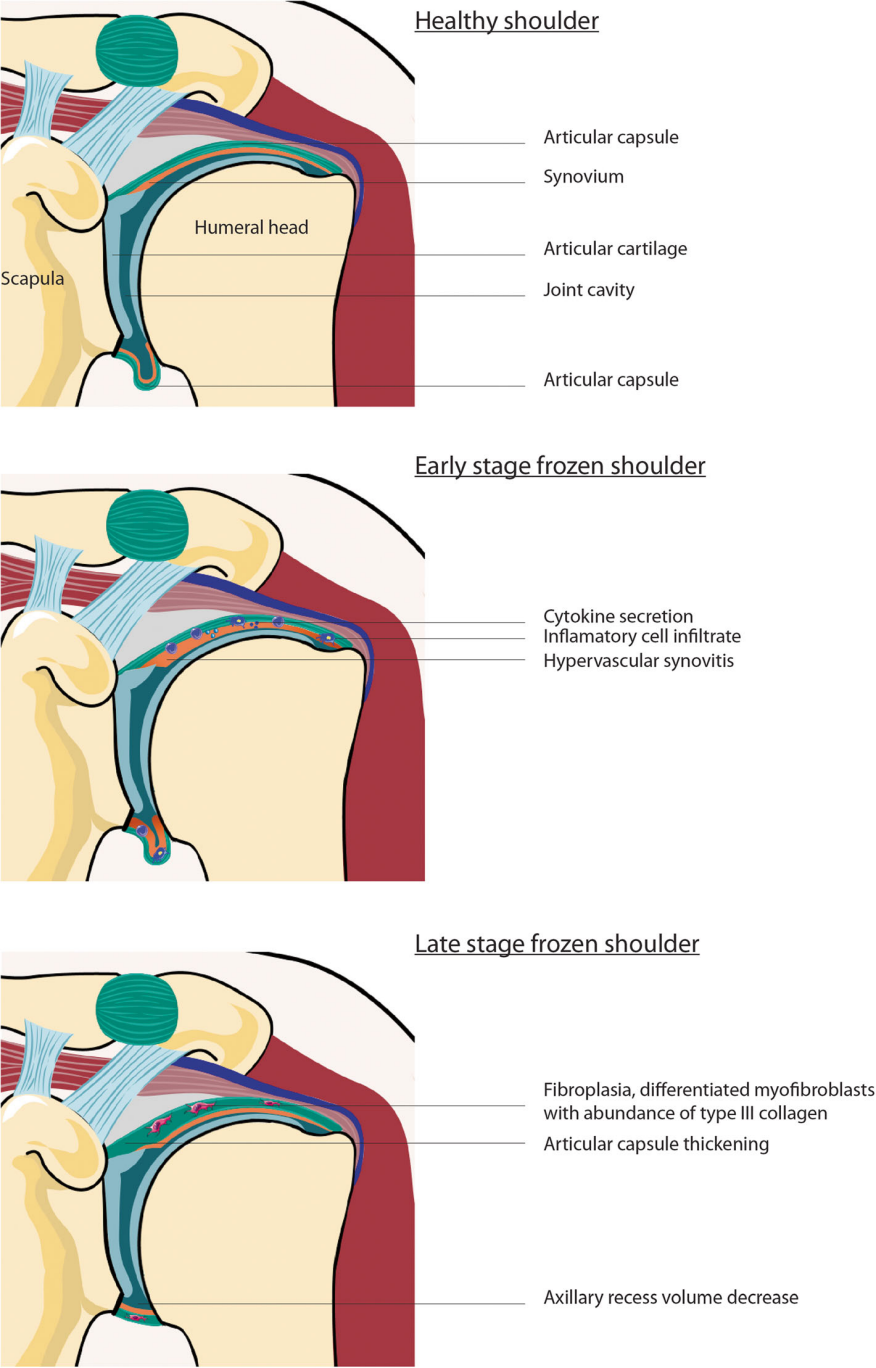
Schematic drawing stages FS

Several authors have studied biopsies of the rotator interval and glenohumeral capsule. Early in the disease process, inflammatory changes with subsynovial hypervascularity, synovial hyperplasia, and fibroblastic proliferation with an increased number of fibroblasts (fibroplasia) is found [5]. This is accompanied by the formation of new nerve fibers around small blood vessels. Neogangionesis is demonstrated by overexpression of hematopoietic cell marker, CD34, and vascular endothelial growth factor (VEGF) [9]. Neurogenesis is driven by an increased expression of nerve growth factor receptor p75 [19]. Besides nerve ingrowth, pro-inflammatory mediators upregulate the acid sensing ion channels that contribute to hyperalgesia [23]. Later on in the disease process, when stiffness is established, the signs of inflammation can disappear gradually [67]. In this stage, an increased number of differentiated fibroblasts into myofibroblasts are seen within an extracellular matrix (ECM) of densely packed disorganized type III collagen [6]. The increased number of contractile myofibroblasts can be picked up with alfa smooth muscle actin (α-SMA) staining, a marker for the differentiation of fibroblasts in myofibroblasts. It has been demonstrated that α-SMA staining is not that prominent yet in the early stage of the disease compared to a more mature FS [25]. To summarize, in the early stage of FS, inflammatory changes can be seen with synovial hyperplasia and subsynovial hypervascularity and neurogenesis. Whereas in the later stage inflammation usually disappears gradually and tissue fibrosis occurs with a high number of fibroblasts within an ECM of densely packed type III collagen. (Fig. 2).

## The mechanism of tissue fibrosis

### Imbalance in extracellular matrix turnover

Fibroblasts are the primary resident cell type in connective tissues. Articular capsule consists of a thin inner synovial lining and an outer layer, which is a more fibrous layer of connective tissue. Fibroblasts are responsible for the production of the ECM, the “soil” in which the cells live and interact. Normally, type I and III collagen are the main proteins in the ECM of normal joint capsule. Type III collagen is the more immature molecule, derived from procollagen [11]. The turnover of ECM is regulated by fibroblasts together with enzymes such as Matrix Metallo Proteinases (MMPs). MMPs degrade abundant collagen and the level of activity of MMPs is counteracted by Tissue Inhibitor of Metallo Proteinases (TIMPs). The fibrotic effects of increased TIMP activity came to light when twelve patients were treated with *Marimastat (British Bio- tech Ltd, Oxford, UK)*, a TIMP analogue for the treatment of gastric carcinoma. Six patients developed bilateral frozen shoulders within four months [68]. The MMP/TIMP ratio has been shown to be almost ten times lower in FS patients versus healthy controls [37]. So, at least a part of the pathophysiologic process leading to fibrosis is a dysregulated collagen synthesis, in other words, an imbalance in ECM turnover.

### Fibroblast contractility: the role of TGF-β1 and mechanical stress

Not only the abundancy of collagen, but also the contractility of fibroblasts in the ECM is a prerequisite to stiffening of the tissue. Myofibroblasts can contract by using a smooth muscle type actin/myosin complex. Vimentin, a cytocontractile protein and marker for contractility, has been shown to be overexpressed in capsular biopsies of FS patients [69]. Interestingly, although fibroplasia has been shown to occur in the entire joint capsule in FS, capsular contracture measured by vimentin staining was more pronounced anteriorly compared to posteriorly [70].

Transforming growth factor-β one (TGF-β1), and mechanical stress are two important factors contributing to contractility of fibroblasts [71]. TGF-β1, an inflammatory cytokine, is present in a lot of tissues throughout the human body, and can be secreted by parenchymal cells, epithelial cells, fibroblasts and by influxing immune cells [72]. The TGF-β1 signaling pathway is believed to have a central role in fibrotic diseases [51, 73]. TGF-β1 has been shown to stimulate contractility of fibroblasts in-vitro collagen gels and can be seen as a potent activator of myofibroblasts [74, 75]. The expression of TGF-β1 and its receptor is increased in biopsies of the joint capsule in FS patients [76]. Besides stimulating myofibroblast differentiation, TGF-β1 also influences ECM turnover by promoting collagen synthesis. Certain genetic variants of genes for the TGF-β pathway and MMPs could be identified as risk factors for the susceptibility of FS [49].

Besides chemical stimulation by cytokines like TGF-β1, mechanical stress is also an important factor in tissue fibrosis. Fibroblasts are mechano-responsive cells, which means that they can ‘sense’ mechanical stress in the ECM with their intracellular cytoskeleton, and their differentiation in to myofibroblasts is stress dependent. In-vitro studies showed that fibroblasts seem to have a threshold for mechanical stress which needs to be reached before they differentiate in to myofibroblasts [77]. Furthermore, mechanical stress has the ability to activate latent TGF-β1, hereby upregulating the process of tissue fibrosis. So, both mechanical stress and TGF-β1 are two important closely interrelated factors in the process of tissue fibrosis [78]. This process is actually a self-reinforcing process. When the tissue gets stiffer, tissue compliance decreases and the mechanical stress recorded by the fibroblasts increases inherently.

## Chronic low-grade inflammation might predispose to the development of FS

Several authors have hypothesized an association with a chronic state of low grade inflammation which might predispose to the development of FS [79]. Several association studies support this theory [38, 40, 48]. Fasting serum cholesterol, triglycerides and plasma glucose levels are often elevated in FS [6, 80]. Inflammatory lipoproteins such as LDL and non-HDL, associated with vascular inflammation and immune reactions, are known risk factors for atherosclerosis. However, these inflammatory lipoproteins have also been identified as independent risk factors for FS [48, 81]. Vascular endothelial cell activation is accompanied by increased expression of intercellular adhesion molecule-1 (ICAM-1), a well-established marker of chronic inflammation. It has also been shown that ICAM-1 levels are elevated in the joint capsule and synovial fluid of FS patients compared to controls [82]. Similar to ICAM-1, is TIMP associated with chronic inflammation. Diabetes mellitus (DM), cardiovascular disorders and thyroid disorders are conditions associated with chronic inflammation and increased levels of similar pro-inflammatory cytokines as are found in FS. This is, at least partially, an explanation why DM and thyroid disorders are strong risk factors for the development of FS, and supports the theory of a chronic state of low-grade inflammation as a predisposing factor in the etiology of FS [83].

## An early inflammatory response at the onset of FS

Traditionally, fibroblasts are known for their structural role in the synthesis and remodeling of ECM in connective tissue. However, fibroblast can also act like sentinel cells involved in immune responses, and thereby modulate the recruitment of immune cells and regulate their behavior [30, 84]. A chronic inflammatory cell infiltrate with mast cells, macrophages, B- and T-cells has been shown to be present in rotator interval biopsies from FS patients [85]. Recent publications suggest that an immune response with an overexpression of inflammatory cytokines is one of the first steps in the development of a FS, preceding the cascade of tissue fibrosis [21, 86]. Cytokines can regulate proliferation, activation and differentiation of fibroblasts, hereby dysregulating collagen synthesis [87]. Multiple studies have shown increased levels of pro-inflammatory cytokines such as TGF-β1, tumor necrosis factor-α (TNF- α), Interleukin-1 and -6 (IL-1, IL-6) and platelet derived growth factor (PDGF) in joint fluid and capsular tissue in FS [7, 21, 86]. Interestingly, increased levels of cytokines were also found in the subacromial bursa in FS patients [21]. When in-vitro cultured fibroblasts are stimulated with joint aspirates of FS patients, fibroblast proliferation was markedly elevated [36]. Furthermore, when fibroblasts were being activated, the inflammatory response was enhanced [88]. A recent study confirmed an elevated level of fibroblast activation markers in capsular tissue biopsies of FS patients compared to controls [30]. Persistent fibroblast activation is a potential cellular mechanism of symptoms of a prolonged frozen stage in FS.

Cytokine release and fibroblast activation is not the first step in the inflammatory response. Capsular biopsies of FS patients have shown elevated levels of several alarmins including High Mobility Group Box 1 (HMGB1) proteins, compared with controls [89]. Alarmins, or Damage-Associated Molecular Pattern (DAMP) molecules, are signal molecules released when cells are distressed, injured or ‘in danger’. Alarmins are the early activators of the immune system and have a role in amplifying the inflammatory response in many inflammatory conditions [90]. HMGB1 can be released into the ECM upon cell death or stress where it mediates an inflammatory reaction. In-vitro cultured human dermal fibroblast and lung fibroblasts stimulated by HMGB1 have been shown to produce more TGF-β1, thereby activating the TGF-β signaling pathway and subsequently significantly upregulate myofibroblast differentiation. And more, HMGB1 has the ability to bind to the receptor of AGE (Advanced Glycation End products) and to activate a pro-inflammatory response through the Nuclear Factor κB (NF-κB) pathway inducing TGF-β1 release [91, 92]. Although an elevated level of alarmins in frozen shoulder capsular biopsies might be quite an aspecific finding, this is an indication that an inflammatory response has an important role at the onset of the pathophysiologic process of FS, triggering the inflammatory cascade leading to tissue fibrosis.

## The implications of  hyperglycaemia in FS

The lifetime prevalence of FS in diabetic patients is with 10–30% much higher than 2–5% in the general population [93–95]. The higher the cumulative hemoglobin A_1c_ level, the higher the incidence of FS [96]. FS tends to be prolonged and more refractory to conservative treatment in diabetics [97]. The exact mechanism behind this is most likely multifactorial. Several authors have hypothesized an important role for AGEs. AGEs are formed by a process called non-enzymatic glycation when glucose forms covalent adducts with proteins, caused by oxidative stress. When AGEs bond to long-lived proteins they cannot be degraded by normal remodeling, and accumulate in connective tissue. This is a normal process which happens progressively with aging, can be slowed down by endurance training, but is accelerated in patients with DM [98]. A particular non-enzymatic ‘AGE’ reaction of interest is the alteration of collagen proteins by crosslinking [26, 99]. Excessive levels of AGEs can lead to pathological collagen crosslinking and structural changes in the tissue, making the tissue less compliant [100]. The level of AGEs has been shown to be significantly higher in capsular tissue samples of FS patients compared to controls [26]. AGEs have also been shown to decrease the expression of MMPs and increasing TIMP expression in diabetic nephropathy, similar to the pathogenic mechanism of imbalance in ECM turnover in FS [101]. And more, it has been shown in diabetic retinopathy and nephropathy that AGEs accumulation can lead to an increased expression of basic fibroblast growth factor and upregulation of the expression of profibrotic cytokines as TGF-β1, PDGF and connective tissue growth factors [102]. It is hypothesized that these pro-fibrotic actions of AGEs also have their role in the pathophysiology of FS, and are part of the explanation why FS in diabetic patients have a tendency to be refractory [26].

## Discussion

**Fig. 3:**
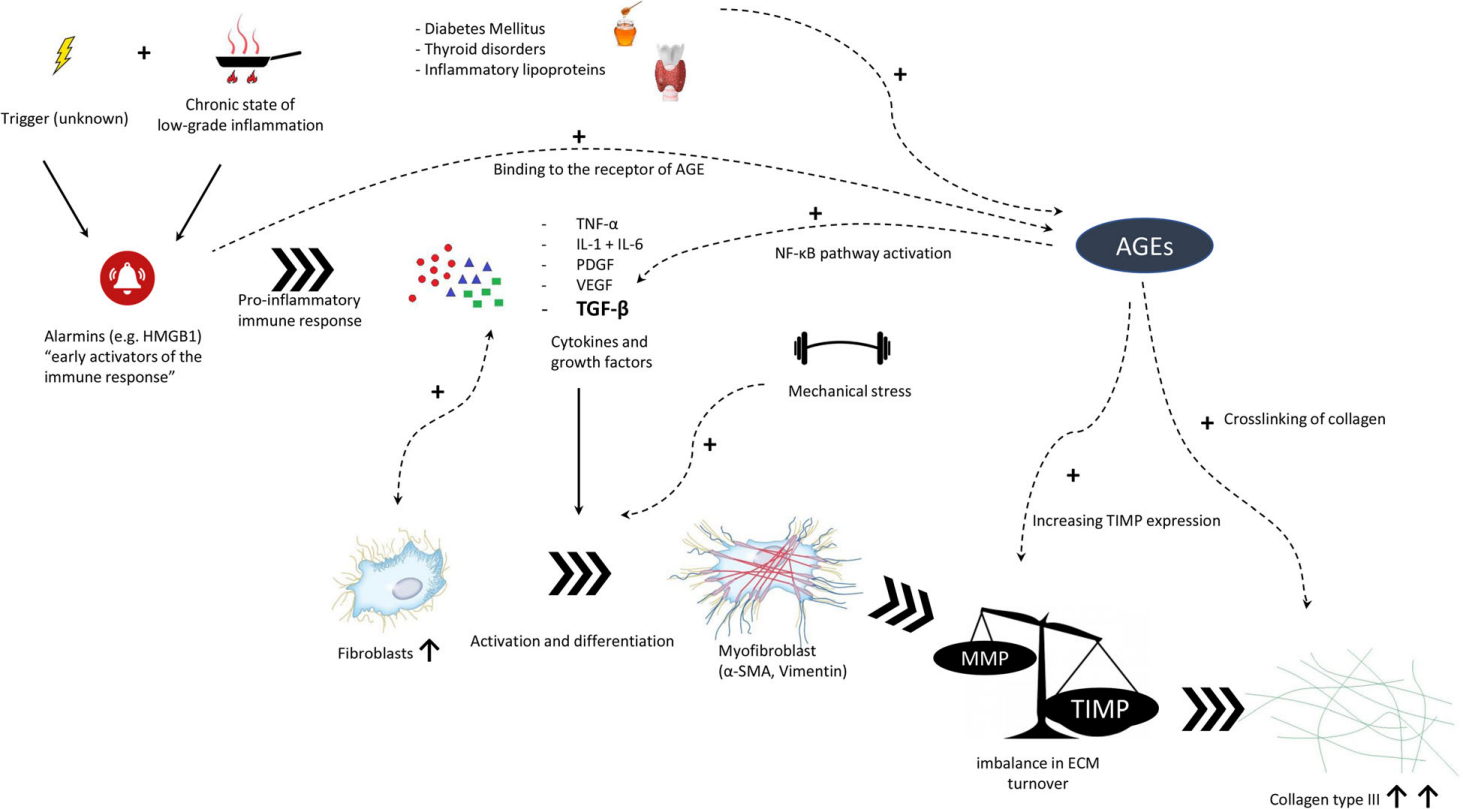
Diagram pathophysiology of FS

It is outlined in this review that the pathophysiology of frozen shoulder is a rather complex process. It involves an early inflammatory response, production of pro-inflammatory cytokines, enhanced fibroblast proliferation, activation and differentiation into myofibroblasts, and an imbalance in ECM turnover with an abundance of disorganized collagen III deposition (Fig. 3). It is clear that there are a lot of factors involved, and we have most likely not identified all related factors yet. There are some important questions that remain unanswered.

### What triggers the onset of a FS?

As with many diseases, it is still unclear what triggers the onset of the disease. Microtrauma has been suggested as a trigger, although this is hard to support with evidence [103]. With the identification of predisposing factors we do get a better understanding of the etiology. An increasing amount of evidence supports a chronic state of low-grade inflammation as an important predisposing factor for the development of FS [46, 48, 79, 81, 104]. Markers of chronic inflammation (ICAM-1, TIMP) are elevated in FS patients, and pro-inflammatory lipoproteins are significant risk factors for FS, similar to patients with cardiovascular disease or metabolic syndrome [48, 82]. The incidence of FS is so much higher in patients with DM and thyroid disorders, since these conditions are associated with a chronic state of inflammation [20, 83]. Even depressive personality traits are sometimes linked to FS, and depression is also associated with enhanced inflammatory cytokine levels [105]. It seems plausible that female hormones might be related in this context, since the peak incidence of FS is in perimenopausal women. However, a clear explanation, or a direct relationship between female hormones and FS was not found in the current literature.

### Why only the shoulder?

How is it possible that FS is a condition unique for the shoulder without similar conditions in other joints? Pietrzak et al. hypothesized an evolutionary explanation [104]. The ability to throw accurately and forcefully is an important ability acquired during human evolution. Therefore, the shoulder is built for elastic energy storage and generation of maximal shoulder external rotation [106]. In our modern sedentary lifestyle without the need for throwing or overhead activities, parts of the anterior shoulder capsule and ligaments are probably not being exercised or stretched sufficiently. This makes the (anterior) shoulder capsule and ligaments probably more susceptible to oxidative stress, related to cytokine production and the formation of AGEs [104]. Although it is uncertain how much of this is true, this could potentially explain why FS is seen less frequently in manual laborers, and why the dominant side seems less likely to be involved [2, 4, 103, 107].

It is debatable whether FS is truly unique to shoulders. Is the capsule of the shoulder so much different to that of other joints? The joint capsule has to be compliant and allows the widest range of motion of all our joints. Is this why shoulder fibroblasts are more ‘sensitive’ to inflammation or mechanical stress? There is some literature about a similar condition in hips, ankles and also knees. However, the currently available literature are mainly case reports of conditions seldomly seen in clinical practice [108, 109]. Contractures with fibrosis do occur frequently mainly in knees and elbows, but without the potential for spontaneous recovery as FS has. We did try to find clues why and how the reversibility happens in FS, but we are not able to find an answer to this question. Apoptosis of the myofibroblasts is probably what occurs in the final stage of the condition, this is how they normally disappear from granulation tissue after wound healing [11, 25].

### Clinical implications and potential future treatment strategies

Physiotherapy and corticosteroids are the most widely used treatment modalities in FS. There is reasonable evidence for the use of intra-articular corticosteroids in the treatment of FS [110]. Corticosteroids have a general suppressive effect on the inflammatory response and hampers the differentiation of fibroblasts into myofibroblasts. Evidence of less α-SMA staining was found, indicating less myofibroblasts, in capsular biopsies in patients treated with corticosteroid injections compared to patients without corticosteroids [25]. One can also understand that the earlier in the disease process the corticosteroid injection is administered, the greater the effect on the clinical symptoms. Corticosteroids can suppress the inflammatory response, but they cannot reverse the fibrotic changes later on in the cascade. When administered in the frozen stage later on, the effect of corticosteroids is usually more temporarily [111].

The negative effect of physiotherapy including mobilization techniques beyond the threshold of pain early on in the disease is explained by the mechanosensitive properties of the fibroblasts [112]. It is hypothesized that the inflammatory response is probably sensitizing the fibroblasts more to mechanical stress. On the other hand, stretching exercises up to a tolerable level of pain resulted in an increase in MMP/TIMP ratio, hereby favoring collagen remodeling and was found to be superior to supervised neglect in the study of Lubis et al. [37] Some mechanical stress is apparently necessary for the remodeling of ECM, especially in the later stage of the condition. This is why tissue irritability, guiding treatment intensity, is implemented in physiotherapy guidelines for the treatment of FS [113].

More advanced treatment strategies have been suggested to intervene with the inflammation-fibrosis cascade in different ways. The TGF-β pathway was interrupted by silencing the Smad4 gene in rats with a FS induced by immobilization, through transfection with a lentivirus [51]. Smad proteins are mediators in the TGF-β signaling cascade. Silencing of this gene suppressed the TGF-β pathway, impairing the inflammatory response and myofibroblast differentiation. The rats with the silenced Smad4 gene had better shoulder range of motion and an increased joint volume compared to rats without Smad4 silencing [51]. Systemic inhibition of TGF-β might have unwanted side effects since it is also an important cytokine for connective tissue homeostasis involved in the proliferation epithelial cells, endothelial cells and immune cells [78]. However, TGF-β inhibitors with low toxicity is a field of intense research. There are now clinical trials with TGF-β inhibitors in cancer patients [114]. Glenohumeral intra-articular infiltration of a TGF-β inhibitor, hereby minimizing systemic effects, could perhaps be a promising suggestion to intervene early on in FS.

Calcitonin was more or less accidentally discovered as a treatment agent for FS when postmenopausal women with FS were treated with calcitonin for osteoporosis [33]. Their FS symptoms improved significantly after the use of a nasal calcitonin spray. Calcitonin is a hormone, secreted by the thyroid, known to inhibit osteoclast activity and lowering the kidney excretion of calcium. The presence of abundant calcitonin receptors in fibroblasts of the shoulder synovium and capsule could be confirmed with immunohistochemistry. Cultured fibroblast from FS patient stimulated with salmon calcitonin showed a significant decrease in the production of collagen type I and III. Synthesis of TGF-beta1 mRNA was suppressed by salmon calcitonin, and the adhesion ability of the fibroblasts decreased with if treated with salmon calcitonin. Apoptosis of the cultured fibroblasts could even be induced with high levels of salmon calcitonin. The efficacy of nasal calcitonin spray was demonstrated in a placebo controlled double blind randomized trial [115]. This might also explain why patients with thyroid disorders have an increased risk of FS, since hypothyroidism and auto-immune thyroiditis can be accompanied by calcitonin deficiency [116, 117].

Intra-articular injections with human recombinant relaxin-2 is suggested as a potential agent for the treatment of FS [52]. Relaxin-2 is known because it is temporarily elevated to soften the cervix during child birth. In an animal study with in vitro cultured fibroblasts Relaxin-2 has been shown to up regulate MMP production, and to down regulate collagen production and expression of TIMP and TGFB-1. This results in a net breakdown of ECM proteins. Furthermore, Relaxin-2 seems to prevent fibroblast differentiation into myofibroblasts. The safety and efficacy still has to be investigated in a human clinical trial. Lee et al. suggested HMGB1 as a therapeutic target and Hinz et al. suggested to target the stress sensors of the fibroblasts, hereby rendering them blind for mechanical stress [78, 91]. However, to what extend these options are realistic and safe options in the near future is unclear.

### Limitations

The search strategy for this scoping review was designed to keep our scope wide to make sure that all available relevant articles are included. A limitation is that the main selection criteria for this scoping review (a substantial focus on pathophysiology of FS) is subjective. Furthermore, the pathophysiologic findings are dependent on the stage of the condition and most of the current research data comes from patients with a refractory frozen stage. To make progress in our understanding of the onset of FS, it might be necessary to include patients early on in the freezing stage in research with histological and immunological analysis.

### Remarks for the future

There are some considerable clinical challenges for healthcare professionals dealing with FS patients. Based on just history and physical examination, it is impossible to predict what the natural course of a FS in an individual patient will be. This is relevant information, not only to inform the patient, but also for shared decision making on when to intervene. Research on prognostic factors for FS is surprisingly scarce. A worse prognosis can be expected in patients with DM and with severe symptoms on presentation [118]. Age over 60 has shown to be a favourable prognostic factor and gender is not correlated with the prognosis [97]. Immunological research seems crucial to get a better understanding of the individual variety in natural history of a FS. Perhaps immune composition in biopsies or biomarkers in synovial fluid can be used as prognostic factors to predict the natural course of FS. Collaboration of orthopedic surgeons with immunologists and rheumatologists is essential in order to move forward in this field of research.

## Conclusions

The complexity of the pathophysiology of FS is outlined in this review. A state of low grade inflammation, as is associated with DM, cardiovascular disease and thyroid disorders, predisposes for the development of FS. An early immune response with elevated levels of alarmins such as HMGB1 and binding to the receptor of AGE starts the cascade of inflammation. Activation of the NF-κB pathway together with mechanical stress stimulates release of inflammatory cytokines, of which TGF-β has a prominent role. Fibroblasts proliferate, become activated and differentiate into myofibroblasts. This results in an imbalance of ECM turnover and a stiff and thickened glenohumeral capsule with abundance of type III collagen. Based on the pathophysiologic mechanism in FS it can be explained why intra-articular corticosteroid injections should be used early on in the condition and why the intensity of physiotherapy should be guided by tissue irritability. Leads are provided to progress with research for clinically important prognostic markers and in search for early interventions in FS.

## Abbreviations

ACIC: Acid sensing ion channel
α-SMA: Alfa smooth muscle actin
AGE: Advanced Glycation End products
CHL: Coracohumeral ligament
DAMP: Damage-associated molecular pattern
DM: Diabetes mellitus
ECM: Extracellular matrix
FDG-PET CT: Fluorodeoxyglucose positron emission tomography/computed tomography
FS: Frozen shoulder
HDL: High density lipoprotein
HMGB1: High mobility group box 1
ICAM-1: Intercellular adhesion molecule-1
IL: Interleukin
LDL: Low density lipoprotein
MMP: Matrix Metallo Proteinases
PDGF: Platelet derived growth factor
RI: Rotator interval
RT-PCR: Real time polymerase chain reaction
TGF-β: Transforming growth factor-beta
TIMP: Tissue Inhibitor of Metallo Proteinases
TNF-α: Tumor necrosis factor alfa
VEGF: Vascular endothelial growth factor
